# Supplementary material for: Association of red blood cell distribution width-to-albumin ratio with mortality in patients undergoing transcatheter aortic valve replacement
Source: PLoS One. 2023 Jun 5;18(6):e0286561. doi: 10.1371/journal.pone.0286561 (PMC10241355; doi:10.1371/journal.pone.0286561)
Supplement: S1 Table — Notes: Cox proportional hazards regression models were used to calculate hazard ratios (HR) with 95% confidence intervals (CI); Model 1 covariates were adjusted for nothing; Model 2 covariates were adjusted for age, gender, hemoglobin, mean corpuscular hemoglobin concentration, blood urea nitrogen, chloride, congestive heart failure, hypertension, atrial flutter/fibrillation, diabetes with complications, and renal disease. RAR, red blood cell distribution width-to-albumin ratio. (DOCX) [file pone.0286561.s001.docx]

**S1 Table. Association of RAR with all-cause mortality after excluding patients who died within 30 days.**

| **Variable** | **Model 1** | | **Model 2** | |
| --- | --- | --- | --- | --- |
|  | **HR (95% CI)** | ***P* value** | **HR (95% CI)** | ***P* value** |
| RAR | 1.70 (1.40~2.07) | < 0.001 | 1.39 (1.08~1.79) | 0.010 |
| RAR Tertile |  |  |  |  |
| < 3.5 | Ref. |  | Ref. |  |
| 3.5-4.0 | 2.17 (1.15~4.10) | 0.017 | 1.51 (0.78~2.93) | 0.226 |
| > 4.0 | 4.59 (2.55~8.26) | < 0.001 | 2.34 (1.19~4.57) | 0.013 |
| *P* for trend |  | < 0.001 |  | 0.009 |

**Notes:** Cox proportional hazards regression models were used to calculate hazard ratios (HR) with 95% confidence intervals (CI); Model 1 covariates were adjusted for nothing; Model 2 covariates were adjusted for age, gender, hemoglobin, mean corpuscular hemoglobin concentration, blood urea nitrogen, chloride, congestive heart failure, hypertension, atrial flutter/fibrillation, diabetes with complications, and renal disease.

RAR, red blood cell distribution width-to-albumin ratio.
