# Supplementary material for: Association of red blood cell distribution width-to-albumin ratio with mortality in patients undergoing transcatheter aortic valve replacement
Source: PLoS One. 2023 Jun 5;18(6):e0286561. doi: 10.1371/journal.pone.0286561 (PMC10241355; doi:10.1371/journal.pone.0286561)
Supplement: Attachment — Submitted filename: Response to Reviewers.docx [file pone.0286561.s003.docx]

Re: Manuscript Number: PONE-D-23-03232

Title: Association of red blood cell distribution width-to-albumin ratio with mortality in patients undergoing transcatheter aortic valve replacement

Dear Editor，

On behalf of my co-authors, we thank you again very much for giving us the opportunity to revise our manuscript. We believe that by implementing the suggested changes, we now have a stronger manuscript entitled “Association of red blood cell distribution width-to-albumin ratio with mortality in patients undergoing transcatheter aortic valve replacement” (Manuscript Number: PONE-D-23-03232) for submission to PLOS ONE.

We have revised the manuscript according to your comments. And we hope the revised manuscript will be acceptable to you. All the changes are indicated in the revised manuscript using track changes. The line and page numbers indicated in our response refer to the revised manuscript.

There are no conflicts of interest regarding this work. All authors have read the revised manuscript and approved its submission to PLOS ONE. Once again, we would like to express our great appreciation to you and reviewers for comments. Looking forward to hearing from you.

Thank you and best regards.

Yours Sincerely,

Guoqiang Gu

Email: [guguoqiang2022@163.com.](mailto:guguoqiang2022@163.com)

Department of Cardiology, The Second Hospital of Hebei Medical University, Shijiazhuang, Hebei, China

Below are our specific responses to the editor’s comments.

**Responses to the Editor:**

**1.** Thank you for your response. Your manuscript has improved after constructive comments from the reviewers. I will not request further analysis. However, there are some simple mistakes. For example, you depicted an arrow in the bar graph in Figure 3 "anemia". Could you please review your manuscript carefully again?

Response 1: Thank you very much for your careful review. We have checked our manuscript carefully. We have remade and reuploaded the Figure 3 according to your suggestion.

Below are our specific responses to the **Journal Requirements**.

**1.** Please review your reference list to ensure that it is complete and correct. If you have cited papers that have been retracted, please include the rationale for doing so in the manuscript text, or remove these references and replace them with relevant current references. Any changes to the reference list should be mentioned in the rebuttal letter that accompanies your revised manuscript. If you need to cite a retracted article, indicate the article’s retracted status in the References list and also include a citation and full reference for the retraction notice.

Response 1: According to the requirements of the journal, we checked for the correctness and formatting of the references and updated them within the manuscript and the reference list. (Page 18, lines 326 and 327, lines 330 and 331)

In addition, we did not cite papers that have been retracted.

We appreciate your efforts in reviewing our manuscript, which have made our study clearer and more comprehensive. We hope that our paper is now acceptable for publication by PLOS ONE.
