# Supplementary material for: Association of red blood cell distribution width-to-albumin ratio with mortality in patients undergoing transcatheter aortic valve replacement
Source: PLoS One. 2023 Jun 5;18(6):e0286561. doi: 10.1371/journal.pone.0286561 (PMC10241355; doi:10.1371/journal.pone.0286561)
Supplement: Attachment — Submitted filename: Response to Reviewers.docx [file pone.0286561.s004.docx]

Re: Manuscript Number: PONE-D-23-03232

Title: Association of red blood cell distribution width-to-albumin ratio with mortality in patients undergoing transcatheter aortic valve replacement

Dear Editor,

Thank you again very much. We have revised the manuscript according to your suggestions. And we hope the revised manuscript will be acceptable to you. All the changes are indicated in the revised manuscript using track changes. The line and page numbers indicated in our response refer to the revised manuscript.

There are no conflicts of interest regarding this work. All authors have read the revised manuscript and approved its submission to PLOS ONE. Once again, we would like to express our great appreciation to you and the reviewers for comments. Looking forward to hearing from you.

Thank you and best regards.

Yours Sincerely,

Guoqiang Gu

Email: [guguoqiang2022@163.com.](mailto:guguoqiang2022@163.com)

Department of Cardiology, The Second Hospital of Hebei Medical University, Shijiazhuang, Hebei, China

Below are our specific responses to the editor’s comments.

**Responses to the Editor:**

**1.** The authors should express gratitude for the administrators of the database MIMIC IV in the Acknowledge section.

Response 1: Thank you very much for your careful review. We have supplemented this part in the revised manuscript. (Page 16, lines 306 and 307)

Below are our specific responses to the **Journal Requirements**.

**1.** Please review your reference list to ensure that it is complete and correct. If you have cited papers that have been retracted, please include the rationale for doing so in the manuscript text, or remove these references and replace them with relevant current references. Any changes to the reference list should be mentioned in the rebuttal letter that accompanies your revised manuscript. If you need to cite a retracted article, indicate the article’s retracted status in the References list and also include a citation and full reference for the retraction notice.

Response 1: According to the requirements of the journal, we checked for the correctness and formatting of the references. In addition, we did not cite papers that have been retracted.

We appreciate your efforts in reviewing our manuscript, which have made our study clearer and more comprehensive. We hope that our paper is now acceptable for publication by PLOS ONE.
